# Viral Suppression Following Switch to Second-line Antiretroviral Therapy: Associations With Nucleoside Reverse Transcriptase Inhibitor Resistance and Subtherapeutic Drug Concentrations Prior to Switch

**DOI:** 10.1093/infdis/jit411

**Published:** 2013-08-13

**Authors:** Victoria Johnston, Karen Cohen, Lubbe Wiesner, Lynn Morris, Johanna Ledwaba, Katherine L. Fielding, Salome Charalambous, Gavin Churchyard, Andrew Phillips, Alison D. Grant

**Affiliations:** 1 Department of Clinical Research, London School of Hygiene and Tropical Medicine, United Kingdom; 2 Division of Clinical Pharmacology, University of Cape Town; 3 The Centre for HIV and Sexually Transmitted Infections, National Institute for Communicable Diseases, Johannesburg,South Africa; 4 Department of Infectious Disease Epidemiology, London School of Hygiene and Tropical Medicine, United Kingdom; 5 Aurum Institute; 6 University of the Witwatersrand, Johannesburg, South Africa; 7 Department of Infection and Population Health, University College London, United Kingdom

**Keywords:** second-line antiretroviral therapy, adherence, resistance, virological failure

## Abstract

***Background.*** High rates of second-line antiretroviral treatment (ART) failure are reported. The association with resistance and nonadherence on switching to second-line ART requires clarification.

***Methods.*** Using prospectively collected data from patients in South Africa, we constructed a cohort of patients switched to second-line ART (1 January 2003 through 31 December 2008). Genotyping and drug concentrations (lamivudine, nevirapine, and efavirenz) were measured on stored samples preswitch. Their association with viral load (VL) <400 copies/mL by 15 months was assessed using modified Poisson regression.

***Results.*** One hundred twenty-two of 417 patients (49% male; median age, 36 years) had genotyping (n = 115) and/or drug concentrations (n = 80) measured. Median CD4 count and VL at switch were 177 cells/µL (interquartile range [IQR], 77–263) and 4.3 log_10_ copies/mL (IQR, 3.8–4.7), respectively. Fifty-five percent (n = 44/80) had subtherapeutic drug concentrations preswitch. More patients with therapeutic vs subtherapeutic ART had resistance (n = 73): no major mutations (3% vs 51%), nonnucleoside reverse transcriptase inhibitor (94% vs 44%), M184V/I (94% vs 26%), and ≥1 thymidine analogue mutations (47% vs 18%), all *P* = .01; and nucleoside reverse transcriptase inhibitor (NRTI) cross-resistance mutations (26% vs 13%, *P* = .23). Following switch, 68% (n = 83/122) achieved VL <400 copies/mL. Absence of NRTI mutations and subtherapeutic ART preswitch were associated with failure to achieve VL <400 copies/mL.

***Conclusions.*** Nonadherence, suggested by subtherapeutic ART with/without major resistance mutations, significantly contributed to failure when switching regimen. Unresolved nonadherence, not NRTI resistance, drives early second-line failure.

The management of first-line, nonnucleoside reverse transcriptase inhibitor (NNRTI)–based antiretroviral treatment (ART) failure is challenging in resource-limited settings. In settings with viral load (VL) monitoring but without resistance testing, guidelines recommend excluding drug interactions and toxicity, intensifying adherence support, and switching to second-line, boosted protease inhibitor (PI)–based ART if a second VL remains elevated [1–3]. This strategy aims to optimize outcomes on second-line ART by ensuring that nonadherent patients with or without drug resistance mutations (DRMs) receive adherence support and that patients with resistance switch regimens in a timely manner.

Nevertheless, under programmatic conditions, high rates of second-line virological failure are reported [4–8]. Delayed switching to second-line ART and consequent accumulation of nucleoside reverse transcriptase inhibitor (NRTI) mutations may contribute [9–14]. Studies indicate that only 17%–53% of patients have switched regimen 12 months following virological failure [9, 15]. Although resistance patterns on identification of virological failure are well described, few data exist on resistance patterns on switching regimens [6, 14, 16] and the influence of nonadherence on such patterns [17–19]. Also, few studies have explored the impact of NRTI mutations and the resultant loss of regimen activity on empirically prescribed second-line ART [4, 16].

Nonadherence on second-line ART is increasingly considered the main driver of early second-line failure [4–7]. Although poor tolerability of PIs may contribute to suboptimal adherence, it is also possible that attempts to intensify adherence support during first-line virological failure were unsuccessful, or not sustained. Measuring the success of adherence interventions is problematic. Healthcare workers' (HCW) assessment and patients' self-report overestimate adherence [20–23], and although drug refill is a reasonable marker of cumulative adherence, it does not measure adherence at a set time-point (eg, following adherence interventions). Alternative markers include the absence of DRMs, or subtherapeutic drug concentrations [24, 25]. Sigaloff et al reported no major DRMs in 12% of patients switching regimens [14]; however, no studies have determined drug concentrations at time of switch.

In a South African treatment program, this study describes the contribution that resistance and nonadherence, as determined by subtherapeutic drug concentrations and the absence of major DRMs, make to first-line virological failure on switching regimens, and investigates the impact of NRTI resistance and nonadherence on response to second-line ART.

## METHODS

### Study Design and Setting

This retrospective cohort analysis used prospectively collected clinic data and stored plasma from patients enrolled in a multisite workplace and community ART program managed by the Aurum Institute, South Africa [26, 27]. Patients were eligible for ART, free of charge, based on World Health Organization clinical staging and CD4 count criteria. First-line ART comprised efavirenz (EFV) or nevirapine (NVP), lamivudine (3TC), and zidovudine (ZDV) or stavudine (d4T). In 2008, tenofovir (TDF) replaced zidovudine (ZDV) in the workplace program. Guidelines recommended a switch to second-line ART (boosted lopinavir [LPV], didanosine [ddI], and ZDV or abacavir [ABC]), if, following adherence counseling, a second VL measurement remained >1000 copies/mL. CD4 count and VL were monitored at baseline and 6-week and 6-month intervals after commencing or switching regimen. One of 2 central laboratories routinely stored excess plasma at −80°C.

### Study Population

Inclusion criteria were age ≥15 years, switched from first- to second-line ART between 1 January 2003 and 31 December 2008, VL >400 copies/mL at switch with available stored plasma (6 months before to 1 week after switch), and potential for at least 15 months of follow-up (data included up to 31 March 2010). Stored samples, from patients with VL >400 copies/mL 12 months (SD, 3 months) following switch, were also analyzed.

### Laboratory Methods

Human immunodeficiency virus (HIV) RNA was assayed using polymerase chain reaction (Amplicor HIV-1 Monitor Test, Roche Diagnostics) and genotyping performed on stored plasma using a modified validated in-house assay [28]. Mutations were identified using the Stanford HIVdb genotypic resistance algorithm (http://hivdb.stanford.edu/) with mixtures reported as mutant genotypes. HIV type 1 (HIV-1) subtype classifications were performed using Rega version 2.0. Plasma drug concentrations were measured using a protein precipitation procedure to extract the drugs, and analyzed using a validated liquid chromatography–tandem mass spectrometry assay. The accuracy and coefficient of variation statistics for 3TC, EFV, NVP, and LPV during sample analysis were 90.7%–108.2% and 1.1%–7.0%, at high, medium, and low quality control concentrations. Limits of quantification were 20 ng/mL for 3TC, 39.1 ng/mL for EFV, and 19.5 ng/mL for NVP and LPV.

#### Outcomes

*Viral suppression on second-line ART* was defined as a VL <400 copies/mL 2 weeks to 15 months following switch. Patients without VL results were excluded if they transferred to another program; otherwise they were considered to have failed treatment. *Alive in care* was defined as no record of leaving the program or loss to follow-up (ie, no contact for ≥6 months) by 15 months.

#### Exposures

Major DRMs were defined using the 2011 update of the International Antiviral Society–USA drug mutations list [29]. Cumulative DRMs were described in patients with >1 sample. *NRTI resistance at switch*, one of the key exposures of interest, was categorized as (1) none, (2) M184V/I, (3) 1–2 thymidine analogue mutations (TAMs) ± M184V/I, and (4) NRTI cross-resistance mutations (≥3 TAMs and/or K65R and/or Q151M and/or T69ins) ± M184V/I.

The second key exposure of interest was *nonadherence at switch*, as determined by subtherapeutic first-line drug concentrations with or without major DRMs. Therapeutic drug monitoring (TDM) guidelines recommend measuring trough drug concentrations (C_trough_) to evaluate efficacy of NNRTIs and PIs, with recommended thresholds for NVP, EFV, and LPV of ≥3 mg/L, ≥1 mg/L, and ≥1 mg/L, respectively [30]. In this study, we assumed that if ART was taken as prescribed, NNRTI and PI concentrations in untimed plasma samples should be greater than C_trough_. Because 3TC plasma concentrations do not correlate well with the active intracellular metabolite, no TDM targets exist. Using data from pharmacokinetic studies of 3TC (300 mg once daily) in plasma, we calculated the population standard deviation and found the lower limit of the 95% confidence interval (CI) for C_trough_ to be approximately 20 ng/mL, the limit of quantification of the assay [31, 32]. Drug concentrations were defined as subtherapeutic if they were greater than C_trough_ (NNRTI and PI) or below the limit of quantification (BLQ) for the assay (3TC), and the regimen as subtherapeutic if either criterion was met. Finally, assuming that subtherapeutic drug concentrations and absence of major DRMs are markers of nonadherence, we categorized nonadherence at switch as therapeutic first-line ART vs subtherapeutic first-line ART plus major DRM vs subtherapeutic first-line ART with no major DRMs.

Patients were categorized to have *reported nonadherence on first-line ART* if, at any visit, the patient self-reported missing ART in the preceding 7 days and/or HCWs reported treatment interruptions for nonadherence. *Duration of viremia on first-line ART*, categorized as <12 and ≥12 months, was defined as the time between the first VL >400 copies/mL following viral suppression to date of switch, where all interim VLs were >400 copies/mL. Only the viremic period immediately preceding switch was considered. For patients without viral suppression on first-line ART, we assumed that (1) patients initiating first-line ART in-program were viremic from this date, and (2) patients transferring into care on ART were viremic for ≥12 months.

### Statistical Analysis

Using risk ratios from modified Poisson regression with robust standard variance, we explored the association between the outcome VL <400 copies/mL on second-line ART and 2 key exposures, NRTI resistance at switch and nonadherence at switch [33]. Potential confounders were derived from prior analyses of this cohort and a literature review [5, 8, 34–36]. Confounders were added sequentially, starting with the variable leading to the greatest degree of confounding, and retained in the final model if they altered the effect size by >10%. Analyses were undertaken using Stata software, version 11.

### Ethical Approval

This study was approved by the research ethics committees of the University of KwaZulu Natal, the University of Cape Town, and the London School of Hygiene and Tropical Medicine. The Aurum Institute program database, maintained for monitoring and evaluation purposes, contained data collected as part of routine clinical care. The workplace employers provided data on reasons for leaving the program through employers' records and hospital death registers. Dates of death were confirmed through program links with the National Death Register. The ethics committees waived the need for patient consent, as data were collected and samples stored as part of routine program practice, and all data, including resistance tests and drug concentrations, were both retrospective and anonymized.

### Genbank Sequence Accession Numbers

KC921018-KC921144

## RESULTS

**Figure 1.**
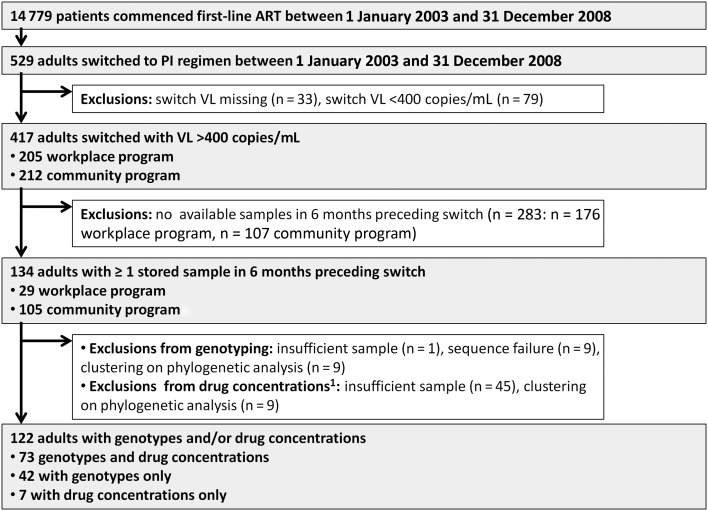
Study flow diagram. Selection of patients for analysis, from a cohort of patients initiating first-line, nonnucleoside reverse transcriptase inhibitor–based antiretroviral therapy between 1 January 2003 and 31 December 2008. Abbreviations: ART, antiretroviral therapy; PI, protease inhibitor; VL, viral load. ^1^Patients with clustering on phylogenetic analysis were also excluded from having drug concentrations measured. One patient had insufficient sample to perform genotyping; however, after genotyping was performed, an additional 44 patients had insufficient samples available for measurement of drug concentrations.

Of 417 patients switched to second-line ART at a VL >400 copies/mL, 134 had available stored samples (n = 29/205 workplace and n = 105/212 community program). Genotyping was successful on 115 patients, of whom 8 had >1 sample genotyped (Figure 1). Nineteen patients were excluded because of sequence failure (n = 9), evidence of clustering on phylogenetic analysis (n = 9), or insufficient sample (n = 1). Eighty patients had sufficient samples for measuring drug concentrations.

The baseline characteristics of patients with and without samples are presented in Table 1 and Supplementary Table 1. One workplace program laboratory did not store samples; therefore, fewer samples were retrieved from this program. The differences in baseline characteristics between patients included and excluded from this study reflect this. The workplace program was predominantly male and the programs used different ART regimens.

**Table 1.** Baseline Characteristics of Patients Switched to Second-line Antiretroviral Therapy (2003–2008) With and Without Available Samples for Genotyping and/or Drug Concentrations

Characteristic	Patients With Samples, No. (%) (n = 122)	Patients Without Samples, No. (%) (n = 295)
Program		
Community	95 (77.9)	117 (39.7)
Workplace	27 (22.1)	178 (60.3)
Age at switch, y, median (IQR)	36 (31–44)	40 (35–48)
Sex, male	60 (49.2)	210 (71.2)
Transfers into program on ART	40/112 (32.8)	80/265 (30.2)
Reason for switch		
Failure	93/111 (83.8)	214/261 (82.0)
Nonadherence	5 (4.5)	10/261 (3.8)
Other	13 (11.7)	37/261 (14.2)
Year of switch		
≤2005	17 (13.9)	37 (12.5)
2006–2007	34 (27.9)	107 (36.3)
2008	71 (58.2)	151 (51.2)
Reported nonadherence, first-line ART	16 (13.1)	46 (15.6)
VL <400 copies/mL, first-line ART	61/98 (62.2)	177/243 (72.8)
Days on first-line ART, median (IQR)	545 (311–810)	601 (393–907)
Duration of viremia		
<12 mo	54/119 (45.4)	123/293 (42.0)
≥12 mo	65/119 (54.6)	170/293 (58.0)
NNRTI preswitch		
EFV	78 (63.9)	220 (74.6)
NVP	44 (36.1)	75 (25.4)
NRTIs preswitch		
ZDV + 3TC	44 (36.1)	182 (61.7)
d4T + 3TC	75 (61.5)	111 (37.6)
Other	3 (2.5)	2 (0.6)
Switch: bPI plus		
ZDV/ddI	59 (48.4)	67 (22.7)
ABC/ddI	36 (29.5)	171 (58.0)
TDF/FTC	9 (7.4)	13 (4.4)
Other	18 (14.7)	44 (14.9)
CD4 count, cells/µL, median (IQR)	177 (77–263)	176 (102–257)
Log_10_ VL, median (IQR)	4.3 (3.8–4.7)	4.5 (4.0–4.9)
Subtherapeutic first-line ART preswitch	44/80 (55)	
HIV subtype (n = 115)		
A	1 (0.9)	
B	2 (1.7)	
C	112 (97.4)	
Resistance mutations		
No major DRM	26 (22.6)	
Any NNRTI mutation	85 (73.9)	
Any NRTI mutation	80 (69.6)	
NRTI, other than M184V/I	42 (36.5)	
NRTI cross-resistance mutations	19 (16.5)	
Two-class resistance	76 (66.1)^a^	

Abbreviations: 3TC, lamivudine; ABC, abacavir; ART, antiretroviral therapy; bPI, ritonavir-boosted protease inhibitor; d4T, stavudine; ddI, didanosine; DRM, drug resistance mutation; EFV, efavirenz; FTC, emtricitabine; HIV, human immunodeficiency virus; IQR, interquartile range; NNRTI, nonnucleoside reverse transcriptase inhibitor; NRTI, nucleoside reverse transcriptase inhibitor; NVP, nevirapine; TDF, tenofovir; VL, viral load; ZDV, zidovudine.

^a^ In 36 of 76 patients, dual class resistance was on the basis of NNRTI mutations and M184V/I alone.

On switching, the median age of patients was 36 years (interquartile range [IQR], 31–44 years), with a median duration on first-line ART of 545 days (IQR, 311–810 days); duration of viremia was 306 days (IQR, 118–547 days), CD4 count was 177 cells/µL (IQR, 77–263 cells/µL), and VL was 4.3 log_10_ copies/mL (IQR, 3.8–4.7 log_10_ copies/mL). Genotyping and/or drug concentrations were measured on samples taken a median of 28 days (IQR, 16–77 days; range 0–153 days) preceding switch. At venous sampling, ART comprised EFV (63.9% [n = 78]) or NVP (36.1% [n = 44]) with the NRTI backbone d4T/3TC (61.5%; n = 75), ZDV/3TC (36.1%; n = 44), TDF/3TC (1.6%; n = 2), or d4T/ddI (0.8%; n = 1). Six patients on ZDV and 2 on TDF had prior exposure to d4T.

### Resistance and Drug Concentrations

Ninety-seven percent (n = 112/115) of patients genotyped were subtype C, 2 were subtype B, and 1 was subtype A. No major DRMs were detected in 23% (n = 26), ≥1 NNRTI mutations were found in 74% (n = 85), and ≥1 NRTI mutations were found in 70% (n = 80), of which M184V/I predominated (67%; n = 77). At least 1 TAM, K65R, or Q151M mutation was detected in 34% (n = 39), 3% (n = 3), and 3% (n = 3) of patients, respectively. Sixteen percent (n = 19) of patients harbored NRTI cross-resistance mutations.

**Figure 2.**
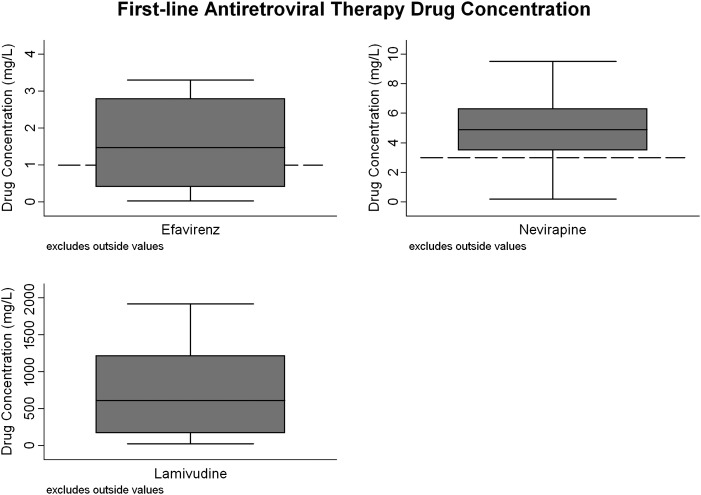
First-line antiretroviral therapy (ART) drug concentrations in stored plasma samples taken prior to switching to second-line ART. Included in this figure are patients with a drug concentration above the limit of quantification of the assay (n = 30/47 patients on efavirenz, n = 23/33 on nevirapine, and n = 44/79 on lamivudine). Excludes outside values: efavirenz, n = 4 (10.9 mg/L, 11.4 mg/L, 14.9 mg/L, 27.6 mg/L); nevirapine, n = 3 (10.6 mg/L, 11.4 mg/L, 17.6 mg/L); lamivudine, n = 0. For each box plot, the median value is denoted by the solid horizontal line; the inter-quartile range by the box; the upper and lower adjacent values by the whiskers; and C_trough_ by the dashed line.

Fifty-six percent (n = 44/79) of patients on 3TC had drug concentrations above the assay's limit of quantification (median, 613 mg/L [IQR, 170–1220 mg/L]; range, 25–1920 mg/L). Fifty-eight percent (n = 19/33) of patients on NVP had drug concentrations greater than C_trough_: 8% (n = 4) 0.01–2.9 mg/L and 30% (n = 10) BLQ. Thirty-eight percent (n = 18/47) of patients on EFV had concentrations greater than C_trough_: 25% (n = 12) <1 mg/L and 36% (n = 17) BLQ (Figure 2). The median drug concentration among patients with detectable NVP and EFV concentrations was 4.91 mg/L (IQR, 3.52–6.31 mg/L; range, 0.19–17.6 mg/L) and 1.47 mg/L (IQR, 0.4–2.8 mg/L; range, 0.02–27.6 mg/L), respectively.

Fifty-five percent (n = 44/80) of patients had subtherapeutic drug concentrations to at least one first-line drug: NNRTI and 3TC were BLQ in 24, NNRTI alone in 8 (NVP subtherapeutic, n = 2; EFV subtherapeutic; n = 4; EFV BLQ, n = 2) and 3TC alone in 11 (all BLQ). One patient whose NVP concentration was BLQ was not on 3TC-containing ART at time of sampling. Data on patients’ self-reported adherence were available for one-third of patients at time of sampling (n = 27/80); 12 of 23 patients reporting adherence had subtherapeutic drug concentrations.

Seventy-three patients had drug concentrations and genotyping performed (Table 2). No major DRMs were detected in 3% (n = 1/34) of patients on therapeutic first-line ART vs 51% (n = 20/39) on subtherapeutic first-line ART (*P* < .01). Major DRMs were more likely to be detected in patients on therapeutic vs subtherapeutic first-line ART: NNRTI mutations in 94% vs 44%, M184V/I in 94% vs 26%, ≥1 TAMs in 47% vs 18% (all *P* ≤ 0.01), and NRTI cross-resistance mutations in 26% vs 13% (*P* = .23), respectively.

**Table 2.** Association Between Resistance and Drug Concentrations on First-line Antiretroviral Therapy (ART) in Samples Taken Prior to Switching to Second-line ART

Resistance Mutations	All	Therapeutic Drug Concentrations on First-line ART^a^	Subtherapeutic Drug Concentrations on First-line ART^b^	
	(n = 73)	(n = 34)	(n = 39)	*P* Value^c^
No major DRM	21 (28.8)	1 (2.9)	20 (51.3)	<.01
Single-class resistance	10 (13.7)	1 (2.9)	9 (23.1)	.02
Two-class resistance	42 (57.5)	32 (94.1)	10 (25.6)	<.01
≥1 NNRTI mutations	49 (67.1)	32 (94.1)	17 (43.6)	<.01
≥1 NRTI mutations	45 (61.6)	33 (97.1)	12 (30.8)	<.01
≥1 NRTI mutations (excluding M184V/I)	26 (35.6)	18 (52.9)	8 (20.5)	.01
M184V/I	42 (57.5)	32 (94.1)	10 (25.6)	<.01
TAM				
0	50 (68.5)	18 (52.9)	32 (82.0)	.01
≥1	23 (31.5)	16 (47.1)	7 (17.9)	
K65R	3 (4.1)	2 (5.9)	1 (2.6)	.6
Q151M	3 (4.1)	3 (8.8)	0 (0.0)	.1
NRTI cross-resistance DRM	14 (19.2)	9 (26.5)	5 (12.8)	.23

Data are presented as No. (%).

Abbreviations: ART, antiretroviral therapy; DRM, drug resistance mutation; NNRTI nonnucleoside reverse transcriptase inhibitor; NRTI, nucleoside reverse transcriptase inhibitor; TAM, thymidine analogue mutation.

^a^ Therapeutic first-line ART: NNRTI concentration equal to or greater than C_trough_ and lamivudine detected.

^b^ Subtherapeutic first-line ART: NNRTI concentration below C_trough_ and/or lamivudine below limit of quantification for the assay.

^c^ Fisher exact test.

### Outcomes on Second-line ART

Eighty-three percent (n = 101/122) of patients were alive in care 15 months following switch to second-line ART (8 died, 7 were lost to follow-up, 4 transferred out, and 2 left for other reasons). There was no difference in retention for those with ≥1 NRTI mutations vs no NRTI mutations (82% [n = 66/80] vs 86% [n = 30/35]; *P* = .67) and those with therapeutic first-line ART preswitch with or without major DRM vs subtherapeutic ART plus major DRM vs subtherapeutic ART with no major DRM (82% [n = 28/34] vs 84% [n = 16/19] vs 75% [n = 15/20], respectively; *P* = .8).

Overall, 68% (n = 83/122) of patients achieved viral suppression on second-line ART. Five patients had no VL results: 4 died or were lost to follow-up and were considered to have failed treatment, whereas 1 patient transferred to another program and was excluded from subsequent analyses. Patients with ≥1 NRTI mutations preswitch were more likely than those without to achieve a VL <400 copies/mL (78% [n = 62/79] vs 51% [n = 18/35]; *P* < .01). Patients on subtherapeutic first-line ART with no major DRMs were less likely to achieve viral suppression than those with major DRMs and those on therapeutic first-line ART (50% [n = 10/20] vs 74% [n = 14/19] vs 85% [n = 28/33], respectively; *P* = .03).

### Association Between NRTI Resistance at Switch and Virological Outcomes

Unadjusted variables associated with viral suppression on second-line ART are presented in Table 3. The presence of NRTI resistance was associated with achieving viral suppression on second-line ART. This remained after adjusting for duration of viremia: cross-resistance DRMs (adjusted relative risk [aRR], 1.87 [95% CI, 1.08–1.71]) vs 1–2 TAMs (aRR, 1.79 [95% CI, 1.23–2.61]) vs M184V/I only (aRR, 1.61 [95% CI, 1.1–2.36]) vs no NRTI mutations (reference); n = 111; *P* = .02. Adjusting for drug concentration on first-line ART, magnitude of viremia, CD4 count, age, calendar year at switch, program, and transfers in on ART did not change the strength of association; these variables were not included in the final model (Table 3).

**Table 3.** Association Between the Key Exposures Nucleoside Reverse Transcriptase Inhibitor Resistance at Switch and Nonadherence at Switch and Our Outcome, Early Viral Suppression on Second-line Antiretroviral Therapy

Key Exposures and Confounders	VL <400 copies/mL / Total (n = 121), No. (%)	Univariable Analysis		Multivariable Analysis			
				NRTI Resistance at Switch (n = 111)		Nonadherence at Switch (n = 70)	
		RR (95% CI)	*P* Value^a^	aRR (95% CI)	*P* Value^a^	aRR (95% CI)	*P* Value^a^
Key exposures							
NRTI resistance at switch	n = 114						
None	18/35 (51.4)	1	.04	1	.02	…	…
M184V/I only	28/38 (73.7)	1.43 (.98–2.09)		1.61 (1.1–2.36)		…	…
1–2 TAMs^b^	19/23 (82.6)	1.61 (1.1–2.33)		1.79 (1.23–2.61)		…	…
Cross-resistance^b^	15/18 (83.3)	1.62 (1.1–2.38)		1.87 (1.24–2.84)		…	…
Nonadherence at switch	n = 72						
Therapeutic ART^c^	28/33 (84.8)	1	.02^d^	…	…	1	.01^d^
Subtherapeutic + DRM	14/19 (73.7)	0.87 (.64–1.18)		…	…	0.85 (.6–1.2)	
Subtherapeutic, no DRM	10/20 (50.0)	0.6 (.37–.94)		…	…	0.53 (.32–.86)	
Confounders				…	…		
Drug concentration at switch	n = 79						
Therapeutic ART	29/35 (82.9)	1	.02			…	…
Subtherapeutic ART	26/44 (29.1)	0.71 (.53–.95)				…	…
Sex						…	…
Male	36/60 (60.0)	1	.05			1	.05
Female	47/61 (77.0)	1.28 (1.0–1.65)				1.39 (1.0–1.93)	
Age at switch							
<35 y	40/52 (76.9)	1	.15				
35–44 y	28/41 (68.3)	0.89 (.69–1.15)					
≥45 y	15/28 (53.6)	0.7 (.48–1.01)					
Duration of viremia	n = 118						
<12 mo	40/53 (75.5)	1.2 (.94–1.52)		1.36 (1.08–1.71)		1.33 (.98–1.8)	
≥12 mo	41/65 (63.1)	1	.15	1	<.01	1	.07
Magnitude of viremia							
Log_10_ ≤4	30/37 (81.1)	1	.06				
Log_10_ VL >4–5	42/62 (67.7)	0.83 (.66–1.05)					
Log_10_ VL >5	11/22 (50.0)	0.62 (.39–.96)					
CD4 count at switch							
<100 cells/µL	22/37 (59.5)	1	.19				
≥100 cells/µL	61/84 (72.6)	1.22 (.91–1.64)					
Year at switch							
≤2007	34/51 (66.7)	1	.7				
2008	49/70 (70.0)	1.05 (.82–1.34)					
Program							
Workplace	11/27 (40.7)	1	<.01				
Community	72/94 (76.6)	1.88 (1.17–3.01)					
Transfers in on ART	n = 111						
No	43/71 (60.6)	1	<.01				
Yes	35/40 (87.5)	1.44 (1.16–1.8)					

Abbreviations: aRR, adjusted relative risk; ART, antiretroviral therapy; CI, confidence interval; DRM, drug resistance mutation; NRTI, nucleoside reverse transcriptase inhibitor; RR, relative risk; TAM, thymidine analogue mutation; VL, viral load.

^a^ Wald test.

^b^ with or without M184V/I.

^c^ All patients had major DRMs detected.

^d^ Test for trend (departure from linear trend *P* > .5).

### Association Between Nonadherence at Switch and Virological Outcomes

After adjusting for confounding due to duration of viremia and sex, patients on subtherapeutic first-line ART without major DRMs were least likely to achieve viral suppression, followed by those with subtherapeutic first-line ART and major DRMs, and finally those on therapeutic ART: subtherapeutic ART, no major DRM (aRR, 0.53 [95% CI, .32–.86]) vs subtherapeutic ART plus major DRM (aRR, 0.85 [95% CI, .6–1.2]) vs therapeutic ART with or without major DRM (reference); n = 70; *P* for trend = .01, *P* for departure from linear trend = .51. Adjusting for magnitude of viremia, CD4 count, age, calendar year at switch, transfers in on ART, and program did not change the strength of association; these variables were not included in the final model.

Of the original cohort of 417 patients, 287 had a VL 12 months following switch to second-line ART, of whom 112 (39%) were >400 copies/mL. Fifteen of 16 samples located were successfully genotyped: 6 patients had no major DRMs, 8 had NNRTI mutations, and 5 had ≥1 NRTI mutation (M184V/I [n = 3], ≥1 TAM [n = 2], K65R [n = 1]). No major PI mutations were detected. Importantly, only 1 of 13 patients in whom drug concentrations were measured had LPV concentrations greater than C_trough_.

## DISCUSSION

This is the first study in a resource-limited setting to determine first-line ART drug concentrations among patients switching to second-line ART. The finding that 55% of patients had subtherapeutic drug concentrations on first-line ART, and that these patients were less likely to achieve viral suppression on second-line ART, suggests that efforts to intensify adherence support when switching regimens were often unsuccessful. Drug concentrations also influenced resistance patterns at switch; NRTI mutations were detected in 97% of patients on therapeutic first-line ART, compared to 31% on a subtherapeutic regimen. However, rather than being associated with virological failure, the presence of NRTI mutations preswitch was associated with achieving viral suppression.

Previous cross-sectional studies of clinic cohorts, which included patients with virological suppression, found that 4%–16% of patients had drug concentrations greater than <C_trough_; however, this is the first study in a resource-limited setting to measure drug concentrations at time of switch [37–39]. The high prevalence of subtherapeutic drug concentrations, at a time when adherence support should have been intensified, underlines the difficulties HCWs encounter in recognizing and successfully addressing nonadherence. Despite 55% of patients having subtherapeutic drug concentrations, nonadherence was the reported reason for switch in only 4%, lower than the 12% reported by Fox et al [5]. This suggests that HCWs failed to recognize and/or report ongoing nonadherence. The lack of pragmatic, valid measures of adherence makes assessing the success of adherence interventions difficult [21]. Certainly, in this study, patients' self-report proved unreliable, with more than half of the patients who reported full adherence having subtherapeutic drug concentrations. Strategies to improve adherence are not guaranteed, as they often require multidimensional, context-specific interventions [23, 40]. Murphy et al, in a South African clinic, found evidence of nonadherence, as measured by drug refill over the 6 months preceding switch, in one-third of patients [7]. Although this improved in the 6 months following switch, the effect was not sustained.

Overall, the pattern of DRMs observed in this study was consistent with others’ findings: no major DRMs in 12% (23% in this study), NNRTIs in 86%–96% (74%), NRTIs in 81%–85% (70%), M184V/I in 68%–74% (67%), and NRTI cross-resistance mutations in 16% (16%) [6, 14, 16]. However, this study demonstrates how subtherapeutic drug concentrations influence resistance patterns. Nearly all patients on therapeutic ART had major DRMs detected, with 26% having NRTI cross-resistance mutations, which could impact on the long-term success of second-line ART. In contrast, half of patients on subtherapeutic first-line ART harbored no major DRMs and 13% harbored NRTI cross-resistance mutations. Although the absence of major DRMs may reflect a true absence due to short periods of nonadherence or very low adherence levels, treatment interruption may result in mutations being BLQ of the assay. For example, K65R, which is thought to emerge more readily in patients with subtype C virus [41], was detected in only 3 of 115 patients. K65R and M184V/I disappear rapidly (<4 months) after treatment interruptions, whereas TAMs, Q151M, and NNRTI mutations persist longer [17–19].

Although the majority of patients achieved early viral suppression on second-line ART (69% in this study vs 77% at 12 months in a meta-analysis [4]), failure to address patients' poor adherence behavior at first-line virological failure, as indicated by subtherapeutic drug concentrations with/without major DRMs, placed them a high risk of failing second-line ART. Patients with subtherapeutic ART and major DRMs preswitch were more likely than those without DRM to achieve viral suppression, suggesting that the presence of major DRMs was acting as an indicator of better past adherence; to have acquired DRMs, patients must have adhered, if suboptimally, to ART. In our study, NRTI mutations did not have a detrimental effect on the activity of second-line ART but instead were associated with viral suppression. Others found no association [4, 16], perhaps due to the potency of PIs, short follow-up period, or small sample size. We believe the positive association found in our study suggests that NRTI mutations are acting as a marker of better past adherence.

Our study has several limitations. First, we have assumed that subtherapeutic drug concentrations are a marker of nonadherence; however, malabsorption or drug interactions may also contribute [42]. One of the commonest drug interactions occurs when both drugs are metabolized by CYP450 (eg, rifampicin and NNRTIs) [43, 44]. 3TC is not metabolized by CYP450 and should not be affected; in this study, 35 of 44 patients with subtherapeutic ART also had undetectable 3TC concentrations. Our definition may misclassify some nonadherent patients as being on therapeutic first-line ART, either because of the untimed nature of the samples or a “white coat adherence” effect, whereby patients take their drugs before attending clinic, resulting in therapeutic drug concentrations despite recent poor adherence. On balance, we believe that subtherapeutic first-line ART (with or without major DRMs) provides a pragmatic, reasonably sensitive marker for nonadherence. Second, not all patients had switch samples, as only one laboratory routinely stored excess plasma and not all stored samples could be located. We do not believe the probability of samples being missing was related to our outcome; therefore, this should not have introduced bias. Third, we were unable to ascertain the respective contribution of resistance and subtherapeutic drug concentrations in all viremic patients on second-line ART. Of 417 patients switched to second-line ART, only 16 of 112 patients with VL >400 copies/mL 12 months following switch had samples available for analysis. The absence of PI mutations in the 15 patients successfully genotyped could be explained by a short follow-up period as PI mutations accumulate at a low rate [45]. However, the majority had low LPV concentrations, making it very likely that nonadherence was a major contributing factor [46–48]. Fourth, our sample size was relatively small, limiting the power to detect associations and assess confounding. Finally, these findings need to be confirmed in other settings. This cohort comprised a workplace- and community-based program. Compared to other programs, the workplace program has been shown to have higher levels of nonadherence and lower levels of viral suppression on first- and second-line ART; however, outcomes in the community-based program, which contributed 80% of this cohort, are comparable to other South African programs [8, 26, 49].

In conclusion, this study highlights that ongoing nonadherence is a major contributor to first-line virological failure when switching regimens and leads to suboptimal second-line outcomes. Patients need intensified adherence support when switching regimens if successful outcomes are to be realized; however, the optimal strategy for achieving this is unclear. In particular, HCWs need better guidance on how to manage patients with first-line virological failure who continue to be nonadherent, despite intensified support. At an individual level, switching nonadherent patients to a boosted PI–based regimen that is less susceptible to development of resistance may be appropriate and is the recommended strategy in many high-income settings. However, the cost-effectiveness of this approach for a public health program, where second- and third-line regimens are costly and treatment options limited, needs to be explored.

## Supplementary Data

Supplementary materials are available at *The Journal of Infectious Diseases* online (http://jid.oxfordjournals.org/). Supplementary materials consist of data provided by the author that are published to benefit the reader. The posted materials are not copyedited. The contents of all supplementary data are the sole responsibility of the authors. Questions or messages regarding errors should be addressed to the author

Supplementary Data

## References

[1] World Health Organization Antiretroviral therapy for HIV infection in adults and adolescents: recommendations for a public health approach. http://www.who.int/hiv/pub/arv/adult2010/en.

[2] Southern African HIV Clinicians Society Guidelines for antiretroviral therapy in adults.. www.sahivsoc.org.

[3] National Department of Health, South Africa Clinical guidelines for the management of HIV and AIDS in adults and adolescents.. http://www.fidssa.co.za/Guidelines/2010_Adult_ART_Guidelines.pdf.

[4] Ajose O, Mookerjee S, Mills EJ, Boulle A, Ford N (2012). Treatment outcomes of patients on second-line antiretroviral therapy in resource-limited settings: a systematic review and meta-analysis. AIDS.

[5] Fox MP, Ive P, Long L, Maskew M, Sanne I (2010). High rates of survival, immune reconstitution, and virologic suppression on second-line antiretroviral therapy in South Africa. J Acquir Immune Defic Syndr.

[6] May Myat W, Maek ANW, Phonrat B, Kiertiburanakul S, Sungkanuparph S (2011). Virologic and immunologic outcomes of the second-line regimens of antiretroviral therapy among HIV-infected patients in Thailand. J Int Assoc Physicians AIDS Care (Chic).

[7] Murphy RA, Sunpath H, Castilla C (2012). Second-line antiretroviral therapy: long-term outcomes in South Africa. J Acquir Immune Defic Syndr.

[8] Johnston V, Fielding K, Charalambous S (2012). Second-line antiretroviral therapy in a workplace and community-based treatment programme in South Africa: determinants of virological outcome. PLoS One.

[9] Johnston V, Fielding KL, Charalambous S, Churchyard G, Phillips A, Grant AD (2012). Outcomes following virological failure and predictors of switching to second-line antiretroviral therapy in a South African treatment program. J Acquir Immune Defic Syndr.

[10] Fox MP, Cutsem GV, Giddy J (2012). Rates and predictors of failure of first-line antiretroviral therapy and switch to second-line ART in South Africa. J Acquir Immune Defic Syndr.

[11] Fox MP, Shearer K, Maskew M (2012). Treatment outcomes after 7 years of public-sector HIV treatment. AIDS.

[12] Cozzi-Lepri A, Phillips AN, Martinez-Picado J (2009). Rate of accumulation of thymidine analogue mutations in patients continuing to receive virologically failing regimens containing zidovudine or stavudine: implications for antiretroviral therapy programs in resource-limited settings. J Infect Dis.

[13] Barth RE, Aitken SC, Tempelman H (2012). Accumulation of drug resistance and loss of therapeutic options precede commonly used criteria for treatment failure in HIV-1 subtype-C-infected patients. Antivir Ther.

[14] Sigaloff KC, Ramatsebe T, Viana R, Wit TF, Wallis CL, Stevens WS (2011). Accumulation of HIV drug resistance mutations in patients failing first-line antiretroviral treatment in South Africa. AIDS Res Hum Retroviruses.

[15] Estill J, Egger M, Johnson LF (2013). Monitoring of antiretroviral therapy and mortality in HIV programmes in Malawi, South Africa and Zambia: mathematical modelling study. PLoS One.

[16] Sigaloff KC, Hamers RL, Wallis CL (2012). Second-line antiretroviral treatment successfully resuppresses drug-resistant HIV-1 after first-line failure: prospective cohort in sub-Saharan Africa. J Infect Dis.

[17] Fox Z, Dragsted UB, Gerstoft J (2006). A randomized trial to evaluate continuation versus discontinuation of lamivudine in individuals failing a lamivudine-containing regimen: the COLATE trial. Antivir Ther.

[18] Gianotti N, Galli L, Boeri E (2005). In vivo dynamics of the K103N mutation following the withdrawal of non-nucleoside reverse transcriptase inhibitors in human immunodeficiency virus-infected patients. New Microbiol.

[19] Trignetti M, Sing T, Svicher V (2009). Dynamics of NRTI resistance mutations during therapy interruption. AIDS Res Hum Retroviruses.

[20] Kouanfack C, Laurent C, Peytavin G (2008). Adherence to antiretroviral therapy assessed by drug level monitoring and self-report in Cameroon. J Acquir Immune Defic Syndr.

[21] Gill CJ, Hamer DH, Simon JL, Thea DM, Sabin LL (2005). No room for complacency about adherence to antiretroviral therapy in sub-Saharan Africa. AIDS.

[22] Murri R, Ammassari A, Trotta MP (2004). Patient-reported and physician-estimated adherence to HAART: social and clinic center-related factors are associated with discordance. J Gen Intern Med.

[23] Thompson MA, Mugavero MJ, Amico KR (2012). Guidelines for improving entry into and retention in care and antiretroviral adherence for persons with HIV: evidence-based recommendations from an International Association of Physicians in AIDS Care panel. Ann Intern Med.

[24] Liechty CA, Alexander CS, Harrigan PR (2004). Are untimed antiretroviral drug levels useful predictors of adherence behavior?. AIDS.

[25] Harrigan PR, Hogg RS, Dong WW (2005). Predictors of HIV drug-resistance mutations in a large antiretroviral-naive cohort initiating triple antiretroviral therapy. J Infect Dis.

[26] Innes C, Hamilton R, Hoffmann CJ (2012). A novel HIV treatment model using private practitioners in South Africa. Sex Transm Infect.

[27] Charalambous S, Grant AD, Day JH (2007). Establishing a workplace antiretroviral therapy programme in South Africa. AIDS Care.

[28] Pillay V, Ledwaba J, Hunt G (2008). Antiretroviral drug resistance surveillance among drug-naive HIV-1-infected individuals in Gauteng Province, South Africa in 2002 and 2004. Antivir Ther.

[29] Johnson VA, Calvez V, Gunthard HF (2011). 2011 update of the drug resistance mutations in HIV-1. Top Antivir Med.

[30] la Porte CJL, Back DJ, Blaschke T (2006). Updated guidelines to perform therapeutic drug monitoring for antiretroviral agents. Rev Antiviral Ther.

[31] Yuen GJ, Lou Y, Bumgarner NF (2004). Equivalent steady-state pharmacokinetics of lamivudine in plasma and lamivudine triphosphate within cells following administration of lamivudine at 300 milligrams once daily and 150 milligrams twice daily. Antimicrob Agents Chemother.

[32] Else LJ, Jackson A, Puls R (2012). Pharmacokinetics of lamivudine and lamivudine-triphosphate after administration of 300 milligrams and 150 milligrams once daily to healthy volunteers: results of the ENCORE 2 study. Antimicrob Agents Chemother.

[33] Zou G (2004). A modified Poisson regression approach to prospective studies with binary data. Am J Epidemiol.

[34] Pujades-Rodriguez M, Balkan S, Arnould L, Brinkhof MA, Calmy A (2010). Treatment failure and mortality factors in patients receiving second-line HIV therapy in resource-limited countries. JAMA.

[35] Lodwick R, Costagliola D, Reiss P (2010). Triple-class virologic failure in HIV-infected patients undergoing antiretroviral therapy for up to 10 years. Arch Intern Med.

[36] Cambiano V, Lampe FC, Rodger AJ (2010). Use of a prescription-based measure of antiretroviral therapy adherence to predict viral rebound in HIV-infected individuals with viral suppression. HIV Med.

[37] Ahoua L, Guenther G, Pinoges L (2009). Risk factors for virological failure and subtherapeutic antiretroviral drug concentrations in HIV-positive adults treated in rural northwestern Uganda. BMC Infect Dis.

[38] van Oosterhout JJ, Bodasing N, Kumwenda JJ (2005). Evaluation of antiretroviral therapy results in a resource-poor setting in Blantyre, Malawi. Trop Med Int Health.

[39] Ferradini L, Laureillard D, Prak N (2007). Positive outcomes of HAART at 24 months in HIV-infected patients in Cambodia. AIDS.

[40] Simoni JM, Amico KR, Smith L, Nelson K (2010). Antiretroviral adherence interventions: translating research findings to the real world clinic. Curr HIV/AIDS Rep.

[41] Wainberg MA, Zaharatos GJ, Brenner BG (2011). Development of antiretroviral drug resistance. N Engl J Med.

[42] Stohr W, Back D, Dunn D (2008). Factors influencing efavirenz and nevirapine plasma concentration: effect of ethnicity, weight and co-medication. Antivir Ther.

[43] Cohen K, van Cutsem G, Boulle A (2008). Effect of rifampicin-based antitubercular therapy on nevirapine plasma concentrations in South African adults with HIV-associated tuberculosis. J Antimicrob Chemother.

[44] Orrell C, Cohen K, Conradie F (2011). Efavirenz and rifampicin in the South African context: is there a need to dose-increase efavirenz with concurrent rifampicin therapy?. Antivir Ther.

[45] Wensing AM, van Maarseveen NM, Nijhuis M (2010). Fifteen years of HIV protease inhibitors: raising the barrier to resistance. Antiviral Res.

[46] van Zyl GU, van Mens TE, McIlleron H (2011). Low lopinavir plasma or hair concentrations explain second line protease inhibitor failures in a resource-limited setting. J Acquir Immune Defic Syndr.

[47] El-Khatib Z, Ekstrom AM, Ledwaba J (2010). Viremia and drug resistance among HIV-1 patients on antiretroviral treatment: a cross-sectional study in Soweto, South Africa. AIDS.

[48] Levison JH, Orrell C, Gallien S (2012). Virologic failure of protease inhibitor-based second-line antiretroviral therapy without resistance in a large HIV treatment program in South Africa. PLoS One.

[49] Dahab M, Charalambous S, Karstaedt AS (2010). Contrasting predictors of poor antiretroviral therapy outcomes in two South African HIV programmes: a cohort study. BMC Public Health.

