# Supplementary material for: Viral Suppression Following Switch to Second-line Antiretroviral Therapy: Associations With Nucleoside Reverse Transcriptase Inhibitor Resistance and Subtherapeutic Drug Concentrations Prior to Switch
Source: J Infect Dis. 2013 Aug 13;209(5):711–20. doi: 10.1093/infdis/jit411 (PMC3923537; doi:10.1093/infdis/jit411)
Supplement: Supplementary Data [file supp_209_5_711__index.html]

Viral suppression following switch to second-line antiretroviral therapy: associations with NRTI resistance and ‘sub-therapeutic’ drug concentrations prior to switch — Viral Suppression Following Switch to Second-line Antiretroviral Therapy: Associations With Nucleoside Reverse Transcriptase Inhibitor Resistance and Subtherapeutic Drug Concentrations Prior to Switch — Viral Suppression Following Switch to Second-line Antiretroviral Therapy: Associations With Nucleoside Reverse Transcriptase Inhibitor Resistance and Subtherapeutic Drug Concentrations Prior to Switch — Supplementary Data 

# Viral Suppression Following Switch to Second-line Antiretroviral Therapy: Associations With Nucleoside Reverse Transcriptase Inhibitor Resistance and Subtherapeutic Drug Concentrations Prior to Switch

## Supplementary Data

Supplementary Data

**Files in this Data Supplement:**

- Supplementary Table 1 - docx file
