# Supplementary material for: Viral Suppression Following Switch to Second-line Antiretroviral Therapy: Associations With Nucleoside Reverse Transcriptase Inhibitor Resistance and Subtherapeutic Drug Concentrations Prior to Switch
Source: J Infect Dis. 2013 Aug 13;209(5):711–20. doi: 10.1093/infdis/jit411 (PMC3923537; doi:10.1093/infdis/jit411)
Supplement: Supplementary Data [file supp_jit411_jit411supp_table1.docx]

**Supplementary material**

**Table S1: Baseline characteristics of patients in workplace and community programme**

|  | | Patients with samples, N (%) | | | Patients without samples, N (%) | | |
| --- | --- | --- | --- | --- | --- | --- | --- |
|  | | **All**  **(N=122)** | ***Workplace (N=27)*** | ***Community (N=95)*** | **All**  **(N=295)** | ***Workplace***  ***(N=178)*** | ***Community***  ***(N=117)*** |
| Age at switch, years  (median, IQR) | | **36 (31-44)** | *44 (36-49)* | *35 (31-44)* | **40 (35-48)** | *42 (37-50)* | *37 (32-43)* |
| Sex, male | | **60 (49.2)** | *26 (96.3)* | *34 (35.8)* | **210 (71.2)** | *162 (91.0)* | *48 (41.0)* |
| Transfers into programme on ART | | **40/112 (32.8)** | *2/26 (7.7)* | *38/86 (44.2)* | **80/265 (30.2)** | *18/159 (11.3)* | *62/106 (58.5)* |
| Reason for switch | |  |  |  |  |  |  |
|  | Failure | **93/111 (83.8)** | *19/24 (79.2)* | *74/77 (96.1)* | **214/261 (82.0)** | *129/156 (82.7)* | *86/105 (81.9)* |
|  | Non-adherence | **5 (4.5)** | *4 (16.7)* | *1 (1.3)* | **10/261 (3.8)** | *9/156 (5.8)* | *0/105 (0)* |
|  | Other | **13 (11.7)** | *1 (4.2)* | *12 (15.6)* | **37/261 (14.2)** | *18/156 (11.5)* | *19/105 (18.1)* |
| Year of switch | |  |  |  |  |  |  |
|  | ≤2005 | **17 (13.9)** | *11 (40.7)* | *6 (6.3)* | **37 (12.5)** | *32 (18.0)* | *5 (4.3)* |
|  | 2006-07 | **34 (27.9)** | *11 (40.7)* | *23 (24.2)* | **107 (36.3)** | *46 (25.8)* | *61 (52.1)* |
|  | 2008 | **71 (58.2)** | *5 (18.5)* | *66 (69.5)* | **151 (51.2)** | *100 (56.2)* | *51 (43.6)* |
| Reported non-adherence, 1^st^-line ART | | **16 (13.1)** | *13 (48.1)* | *3 (3.2)* | **46 (15.6)** | *41 (23.0)* | *5 (4.3)* |
| VL <400 copies/ml, 1^st^-line ART | | **61/98 (62.2)** | *12/25 (48.0)* | *49/73 (67.1)* | **177/243 (72.8)** | *118/165 (71.5)* | *59/78 (75.6)* |
| Days on 1^st^-line ART  (median, IQR) | | **545 (311-810)** | *620 (376-805)* | *536 (295-818)* | **601 (393-907)** | *705 (449-1031)* | *510 (306-691)* |
| Duration of viraemia | |  |  |  |  |  |  |
|  | <12 months | **54/119 (45.4)** | *11/27 (40.7)* | *43/92 (46.7)* | **123/293 (42.0)** | *71/178 (39.9)* | *52/115 (45.2)* |
|  | ≥12 months | **65/119 (54.6)** | *16/27 (59.3)* | *49/92 (53.3)* | **170/293 (58.0)** | *107/178 (60.1)* | *63/115 (54.8)* |
| NNRTI pre-switch | |  |  |  |  |  |  |
|  | EFV | **78 (63.9)** | *24 (88.9)* | *54 (56.8)* | **220 (74.6)** | *164 (92.1)* | *56 (47.9)* |
|  | NVP | **44 (36.1)** | *3 (11.1)* | *41 (43.2)* | **75 (25.4)** | *14 (7.9)* | *61 (52.1)* |
| NRTIs pre-switch | |  |  |  |  |  |  |
|  | AZT+ 3TC | **44 (36.1)** | *26 (96.3)* | *18 (18.9)* | **182 (61.7)** | *164 (92.1)* | *18 (15.4)* |
|  | d4T+ 3TC | **75 (61.5)** | *1 (3.7)* | *74 (77.9)* | **111 (37.6)** | *12 (6.7)* | *99 (84.6)* |
|  | Other | **3 (2.5)** | *0* | *3 (3.2)* | **2 (0.6)** | *2 (1.2)* | *0* |
| Switch: bPI + | |  |  |  |  |  |  |
|  | AZT/ddI | **59 (48.4)** | *0* | *59 (62.1)* | **67 (22.7)** | *1 (0.6)* | *66 (56.4)* |
|  | ABC/ddI | **36 (29.5)** | *25 (92.6)* | *11 (11.6)* | **171 (58.0)** | *161 (90.4)* | *10 (8.5)* |
|  | TDF/FTC | **9 (7.4)** | *0* | *9 (9.5)* | **13 (4.4)** | *2 (1.1)* | *11 (9.4)* |
|  | Other | **18 (14.7)** | *2 (7.4)* | *16 (16.8)* | **44 (14.9)** | *10 (5.6)* | *29 (24.7)* |
| CD4 count, cells/mm^3^  (median, IQR) | | **177 (77-263)** | *157 (58-198)* | *185 (89-295)* | **176 (102-257)** | *172 (102-237)* | *188 (110-269)* |
| log_10_ VL (median, IQR) | | **4.3 (3.8-4.7)** | *4.6 (4.2-5)* | *4.3 (3.7-4.6)* | **4.5 (4.0-4.9)** | *4.6 (4.1-5.1)* | *4.2 (3.8-4.6)* |
| ‘Sub-therapeutic’ 1^st^-line ART pre-switch | | **44/80 (55)** | *11/15 (73)* ^1^ | *33/65 (51)* ^1^ |  |  |  |
| HIV subtype | | **N=115** | *N=27* | *N=88* |  |  |  |
|  | A | **1 (0.9)** | *0* | *1 (1.1)* |  |  |  |
|  | B | **2 (1.7)** | *0* | *2 (2.3)* |  |  |  |
|  | C | **112 (97.4)** | *27 (100.0)* | *85 (96.6)* |  |  |  |
| No major DRM | | **26 (22.6)** | *8 (29.6)* | *18 (20.5)* |  |  |  |
| Any NNRTI mutation | | **85 (73.9)** | *18 (66.7)* | *67 (76.1)* |  |  |  |
| Any NRTI | | **80 (69.6)** | *16 (59.3)* | *64 (72.7)* |  |  |  |
| NRTI, other than M184V/I | | **42 (36.5)** | *9 (33.3)* | *33 (37.5)* |  |  |  |
| NRTI cross-resistance mutations | | **19 (16.5)** | *6 (22.2)* | *13 (14.8)* |  |  |  |
| Two class resistance | | **76 (66.1)^a^** | *15 (55.6)* | *61 (69.3)* |  |  |  |

^a^in 36/76 patients’ dual class resistance was on the basis of NNRTI mutations and M184V/I alone

^1^p=0.15

**Abbreviations:** IQR, inter-quartile range; NNRTI, non-nucleoside reverse transcriptase inhibitor; NRTI, nucleoside reverse transcriptase inhibitor; bPI, ritonavir boosted protease inhibitor; VL, viral load; DRM, drug resistance mutation.
